# Graded Exercise Testing Protocols for the Determination of VO_2_max: Historical Perspectives, Progress, and Future Considerations

**DOI:** 10.1155/2016/3968393

**Published:** 2016-12-25

**Authors:** Nicholas M. Beltz, Ann L. Gibson, Jeffrey M. Janot, Len Kravitz, Christine M. Mermier, Lance C. Dalleck

**Affiliations:** ^1^Health, Exercise & Sports Sciences Department, University of New Mexico, Albuquerque, NM, USA; ^2^Department of Kinesiology, University of Wisconsin-Eau Claire, Eau Claire, WI, USA; ^3^Recreation, Exercise & Sports Science Department, Western State Colorado University, Gunnison, CO, USA

## Abstract

Graded exercise testing (GXT) is the most widely used assessment to examine the dynamic relationship between exercise and integrated physiological systems. The information from GXT can be applied across the spectrum of sport performance, occupational safety screening, research, and clinical diagnostics. The suitability of GXT to determine a valid maximal oxygen consumption (VO_2_max) has been under investigation for decades. Although a set of recommended criteria exists to verify attainment of VO_2_max, the methods that originally established these criteria have been scrutinized. Many studies do not apply identical criteria or fail to consider individual variability in physiological responses. As an alternative to using traditional criteria, recent research efforts have been directed toward using a supramaximal verification protocol performed after a GXT to confirm attainment of VO_2_max. Furthermore, the emergence of self-paced protocols has provided a simple, yet reliable approach to designing and administering GXT. In order to develop a standardized GXT protocol, additional research should further examine the utility of self-paced protocols used in conjunction with verification protocols to elicit and confirm attainment of VO_2_max.

## 1. Brief History of Graded Exercise Testing

The examination of the dynamic human physiological responses during incremental exercise has been an ever-evolving task for nearly 200 years. Beginning as early as the 18th century and continuing through the 19th century, pioneering physiologists such as Antoine Lavoisier and Nathan Zuntz have been credited with the first scientific examinations involving exercising humans under normal and hypoxic conditions. In 1918, Lambert described the use of a series of exercise tests to examine the impact on blood pressure to establish a reliable index of myocardial efficiency [1]. Inspired by Lambert and the foundational works of Francis Benedict, Goran Liljestrand, and August Krogh, British physiologist Archibald Vivian (A. V.) Hill conducted a fundamental series of experiments that remain the genesis of exercise physiology as an academic discipline [2]. Using Douglas bags to collect expired air samples, Haldane gas analyzers to determine fractional concentrations of oxygen and carbon dioxide, and a Tissot gasometer to measure air volumes, Hill and colleagues [3–6] repeated running trials of increasing speeds to plot the relationship between intensity and oxygen uptake (VO_2_). Interestingly, it was concluded that a “ceiling” or upper limit in the maximal uptake of oxygen (VO_2_max) existed [7]. It must be appreciated that a difference exists between VO_2_peak and VO_2_max and that these terms are often used interchangeably in the literature. That is, VO_2_peak is the highest value attained during exercise and represents an individual's exercise tolerance while VO_2_max represents the highest physiologically attainable value [8]. Interestingly, a VO_2_max is always a peak but a VO_2_peak is not always maximal. The difference between VO_2_peak and VO_2_max is often determined by the presence of a VO_2_ “plateau,” although the plateau depends on many protocol variables. This review will further expound on this point. Hill's experiments and a century of observations before him led to the discovery of graded exercise testing (GXT), the gold standard for quantifying cardiorespiratory fitness (CRF) and measuring VO_2_ during incremental to maximal exercise.

## 2. Applications of GXT

Graded exercise testing is used to observe the dynamic relationship between exercise workload and the integrated cardiovascular, pulmonary, musculoskeletal, and neuropsychological systems [9]. Protocols require a systematic and linear increase in exercise intensity over time until the individual is unable to maintain or tolerate the workload. Selected cardiovascular, pulmonary, and metabolic variables are collected during the test to evaluate exercise tolerance and represent the efficiency in which the cardiovascular system is able to deliver oxygenated blood to working skeletal muscle and the ability of muscle to utilize oxygen. Due to the widespread use of GXT in healthy populations, normative criteria have been established to help practitioners identify metabolic and ventilatory patterns. Moreover, these metabolic and ventilatory patterns may even assist in categorizing cardiovascular disease (CVD) states and prognoses [9].

The assessment of exercise tolerance has been used to establish the relationship between CRF, CVD, and all-cause mortality [10]. An early investigation by Blair et al. [10] examined the relationship between fitness and mortality in 32,421 men and women (20–80 years old) encompassing 264,978 living-years and 690 deaths. Their results were in agreement with a previous investigation [11] that found substantial strength and independence of low CRF as a predictor for all-cause mortality and future CVD. Similarly, a meta-analysis by Kodama et al. [12] showed that a 1-MET (3.5 mL·kg^−1^·min^−1^) increase in VO_2_max was associated with a 13% and 15% reduction in risk of all-cause mortality and CVD, respectively. Furthermore, a threshold to classify a substantially higher risk for all-cause mortality and CVD was established as low CRF (<7.9 METS) [12]. The evidence clearly demonstrates the influence of low CRF as an independent precursor to mortality and underpins the application of standardized protocols to monitor VO_2_max in specific populations. These studies highlight the importance of accurate and reliable GXT results for minimal exercise tolerance and clinical evaluation of health status [10–12]. Furthermore, valid GXT results are relevant when interpreting studies using repeated measurements of VO_2_max (and not VO_2_peak) to establish a training effect or design exercise prescription.

Accurately capturing the dynamic physiological responses during GXT is essential to establish a valid VO_2_max and to quantify CRF responses throughout various training interventions. Many independent factors contribute to the varying opinions surrounding the appropriateness of current standardized GXT guidelines, thereby limiting the ability to compare results between tests and population or apply them. The modes for administering GXT are traditionally limited to cycle and treadmill, each resulting in unique physiological responses. Protocol design variables such as stage length, workload increment per stage, and total test duration may individually limit the accuracy of GXT. Furthermore, the criteria used to confirm attainment of VO_2_ plateau are not consistent or universally applied among studies. This review will examine the limitations within the current recommendations for GXT and highlight the importance of the continuous search for identifying an optimal protocol.

## 3. Applications of the Fick Equation

The importance of seminal works by Hill et al. [4–6] is apparent considering that the fundamental basis for quantifying oxygen transport, utilization, and mitochondrial energy production remain the same today as they did nearly 100 years ago. The Fick equation states that VO_2_ is equal to the product of cardiac output (*Q*) and the difference between arterial and venous oxygen content at the level of the capillary (a-vO_2diff_).

(1) VO2mL·kg−1·min−1=QL·min−1×a-vO2diffmL·L−1.

The equation can be expanded to represent *Q* as the product of heart rate (HR) and left ventricular (LV) stroke volume (SV), with SV being parsed into the difference between LV end-diastolic volume (EDV) and end-systolic volume (ESV).

(2) VO2mL·kg−1·min−1=HRb·min−1·EDVmL·b−1−ESVmL·b−1a-vO2diff·mL·L−1.

Altogether, the components of Fick represent individual central (*Q*) and peripheral (a-vO_2diff_) factors. The central component consists of factors that impact the diffusion of O_2_ from the external environment into the arterial blood supply and transport of oxygenated blood to working skeletal muscle tissue. The peripheral component comprises various cellular and molecular mechanisms at the skeletal muscle level to diffuse O_2_ from arterial blood to the mitochondria for consumption in the process of ATP regeneration [13].

### 3.1. *Q* Implications of the VO_2_max Protocol

It has been well established that *Q* increases at a linear rate similar to VO_2_ upon the initiation of incremental to maximal exercise [14–18]. In response to metabolically induced peripheral vasodilation in working skeletal muscle, blood pressure is maintained by increases in HR and SV [16]. Central medullary control of baroreceptors, chemoreceptors, and vascular tone contributes to the withdrawal of parasympathetic activity coupled with an increase in sympathetic drive. The result is an overall increase in chronotropic and inotropic characteristics of the heart.

Generally, HR increases linearly during incremental to maximal exercise; however, a breaking point (i.e., HR threshold) is eventually obtained after which the slope may increase or decrease until maximal heart rate (HRmax). The HR threshold is an individual phenomenon that may indicate chronotropic insufficiency [19]. Moreover, the significance of the flattened HR response following HR threshold, in particular, may be associated with a downregulation in beta-1 adrenergic receptor activation during greater exercise intensities. For instance, a study by Knight-Maloney et al. [19] examined HR responses during incremental to maximal exercise in 14 healthy individuals and found that eight subjects demonstrated a decelerated post-HR threshold response while six subjects showed an accelerated post-HR threshold response. These findings are in agreement with previous research that demonstrated the intersubject variability in HR responses [20–24].

Alongside the increasing inotropic response, shifts in sympathetic nervous system dominance raise chronotropic activity and influence central mechanical changes during incremental to maximal exercise. Neural drive enhances myocardial contractility, reducing ESV. Additionally, the intramuscular oscillations during exercise promote an increase in venous return to the heart. The improved blood flow to the LV enhances preload and promotes LV myocardial stretch, increasing elastic potential energy for additional contractile force. This is known as the Frank-Starling mechanism [25]. The net effect is an increase in SV, contributing to an increase in *Q* during incremental to maximal exercise. Contrary to traditional thought, SV within a healthy population may exhibit individual linear or plateau responses that are dependent on many factors.

Pioneering work by Astrand et al. [26] evaluating the SV response to incremental to maximal exercise established the widely accepted observation that SV plateaus are at approximately 40–50% of VO_2_max [27, 28]. This finding was primarily attributed to tachycardiac limitations on diastolic filling time, therefore reducing EDV and blunting SV response [29]. More recently, a review by Vella and Robergs [30] underscores that the potential determinants of the intersubject variability in SV are more complex than originally established. Factors such as age, fitness level, and sex contribute to four main SV responses: the classic plateau, plateau with subsequent drop, plateau with subsequent rise, and gradual increase. While some studies have reported linear SV responses during incremental to maximal exercise in older individuals [31, 32] other studies showed that SV exhibited either a plateau or a subsequent drop at nonspecific points during incremental to maximal exercise [18, 33–35]. Although it may be logical to suggest that age has a negative impact on the maintenance of SV near maximal exercise due to reductions in myocardial compliance, the overall relationship between age and SV response remains unclear. Similarly, individual fitness level does not reliably predict the trend in SV during exercise. Some investigations showed a constant increase in SV up to maximal [36–39] and near maximal intensities [40] in trained individuals while others reported a plateau in both trained and untrained subjects [32, 41] and progressive increases until maximal exercise in untrained subjects [42–44]. One could postulate that adaptations consequent to aerobic training enhance SV through combinations of the following: increased blood volume leading to greater EDV, increased LV chamber size, improved LV compliance, greater myocardial contractility, and reduced afterload may explain the individual ability to increase SV progressively through incremental to maximal exercise [30].

### 3.2. A-vO_2diff_ Implications of the VO_2_max Protocol

It has also been accepted that an increase in *Q* was the sole component of maintaining VO_2_ during the onset of exercise due to a potential lag between oxygen demand and venous return [45–47]. This was tested by Casaburi et al. [48], who found that pulmonary artery desaturation occurred as soon as four seconds after the onset of 150-Watt cycle exercise. The results reported by the Casaburi et al. [48] were questioned by De Cort et al. [45] and attributed to immobilized vena caval blood. Compared to Casaburi et al. [48], who measured a-vO_2diff_ upon exercise from rest, De Cort et al. [45] began measurements starting at the first increase in VO_2_ after the abrupt increase in cycling intensity from a submaximal level. Although a-vO_2diff_ may improve with aerobic training [49, 50], the cellular mechanisms contributing to oxygen extraction within skeletal muscle also increase at a predictable rate during incremental to maximal exercise. Recent meta-analyses by Montero et al. [51] and Montero and Diaz-Canestro [52] examined the effects of aerobic training on a-vO_2diff_ in untrained or moderately trained healthy young (<40 years old), middle-aged, and/or older (≥40 years old) individuals. These studies concluded that the improvements in VO_2_max from 5 to 52 weeks of endurance training were due to linear improvements in *Q*_max_ but not a-vO_2diff_. Therefore, the peripheral mechanisms are viewed as a complement to the central mechanisms contributing to VO_2_max.

## 4. VO_2_max Protocol Critical Considerations

The foundational study by Taylor et al. [53] demonstrated that the sensitivity and reliability of physiological responses during GXT were limited by subject characteristics and GXT protocol design. In an attempt to investigate this issue, 115 healthy males (18–35 years) completed a wide range of GXT protocols under various conditions of physical stressors (caloric restriction, bed rest, temperature, and illness). It was the first comprehensive study to examine the sensitivity and reliability of VO_2_max based on modality, fitness status, illness, environment, gas sampling rate, test duration, and speed/grade increments.

Since then, there has been a search for an optimal standardized protocol suitable for the entire spectrum of fitness abilities and testing goals. The two modalities commonly used in GXT are treadmill and cycle ergometry. While the treadmill appears to be the most widely used modality due to familiarity with upright locomotion and greater muscle mass utilization, cycling protocols present an opportunity to test individuals with coordination or orthopedic limitations. Furthermore, opting to use a cycle ergometer over treadmill may result in a more quantifiable workload (Watts) and provides an opportunity to use a progressive ramp protocol allowing for more reproducible outcomes [9]. However, VO_2_max attained using treadmill protocols tend to produce up to 20% greater VO_2_max values when compared to cycle protocols [54, 55]. This difference is attributed to a larger recruitment of exercising skeletal muscle mass, *Q* and a-vO_2diff_, vascular conductance, and a lower rate of carbohydrate oxidation leading to a less severe development of metabolic acidosis at submaximal intensities [15, 56–60].

Realizing the need to investigate physiological responses to the earliest standardized GXT protocols, Pollock et al. [61] compared cardiopulmonary responses between four widely used treadmill testing protocols in 51 men (22 active, 29 sedentary): Balke [62], Bruce [63], Ellestad [64], and modified Astrand [65]. Each test differed in the method of increasing work rate in a step fashion (either speed or grade). The Balke protocol maintains a constant speed (3.3 mph) but increases grade by 1% each minute. The Bruce protocol increases speed and grade every 3 min. The Ellestad protocol increases speed each stage until the 10th minute upon introduction of a single increase in grade (to 5%) followed by subsequent increases in speed. Finally, the Astrand protocol maintains a constant running speed with increase in grade (2.5%) every 2 min. Pollock et al. [61] observed a similar VO_2_max achieved between Balke, Bruce, Ellestad, and Astrand protocols (39.4, 40.0, 40.7, and 41.8 mL·kg^−1^·min^−1^, resp.) despite the difference in VO_2_ plateau attainment (69%, 69%, 59%, and 80% of participants, resp.). Interestingly, this shows that the individual characteristics of similar protocols do not impact VO_2_max but show inconsistencies in plateau. Their finding was one of the first to demonstrate the impact of protocol design characteristics on the attainment of VO_2_max and a VO_2_ plateau.

Early investigations by Whipp et al. [47] and Davis et al. [66] popularized the use of ramp protocols on electronically braked cycle ergometers. It was proposed that ramp cycle protocols would improve an individual's ability to reach VO_2_max because the ramp increased work in a much more continuous fashion when compared to step increases in work rate used in traditional treadmill protocols [67]. Since workload was deliverable in a linear fashion, attention turned to examining the slope of the VO_2_-work rate (ΔVO_2_/ΔWR) relationship. Buchfuhrer et al. [67] compared cycle tests of various changes in work rates (15 W·min^−1^, 30 W·min^−1^, and 60 W·min^−1^) and noted that ramping at an intermediate rate (30 W·min^−1^) produced the greatest VO_2_max values; however, the work rate was dependent on fitness status. In a similar study, Zhang et al. [68] compared multiple work rates (15 W·min^−1^, 20 W·min^−1^, and 30 W·min^−1^) applied in continuous ramp versus step (1 min, 2 min, and 3 min) fashion. Interestingly, no differences were found in aerobic parameters (VO_2_max, anaerobic threshold (AT), AT/VO_2_max, and ΔVO_2_/ΔWR) between any of the protocols. Furthermore, this study emphasized the importance of work rate increments independent of the stage length used. In contrast, Myers et al. [54] showed that ramp protocols (treadmill and cycle) represented a higher correlation between VO_2_ and workload compared to step, thus reducing the error in predicting the metabolic cost at individual workloads. Muscat et al. [55] completed the most comprehensive investigation to date, comparing physiological responses (cardiometabolic function, gas exchange, breathing patterns, pulmonary function, and leg discomfort) between ramp treadmill and ramp cycle protocol matched for work increase (25 W/2 min) in 15 healthy young men. It was concluded that VO_2_, VCO_2_, respiratory exchange ratio (RER), HR, O_2_ pulse, ventilation, and respiratory muscle effort (diaphragm) responses were greater at maximal and submaximal workloads for treadmill compared to cycle exercise; however, the responses in ventilatory equivalents and ventilatory thresholds were similar. Their results suggested that either mode may be applied for purposes of evaluating mode-specific fitness and determining optimal training prescriptions when work rate increases are applied in a ramp fashion.

Similar to protocol mode (cycle versus treadmill), stage length, and work rate increments (ramp versus step), GXT protocol duration should be considered when comparing results. Buchfuhrer et al. [67] utilized 1-minute stage protocols to examine the impact of protocol duration on the achievement of VO_2_max. Since VO_2_max values were greater in protocols lasting between 8 and 17 min compared to tests outside these limits, the current duration recommendation of 8–12 min was established [67]. More recently, Yoon et al. [69] suggested that the Buchfuhrer et al. [67] study lacked appropriate statistical power and commented that overall test duration (5–14 min) may depend on age and training status [70–72]. Yoon et al. [69] compared VO_2_max and incidence of VO_2_ plateau across four protocols of different durations (5, 8, 12, and 16 min) using a cycle ramp protocol in moderate-to-highly trained individuals. They found that VO_2_max was higher in men for the 8-minute protocol compared to 5-, 12-, and 16-minute protocols, while there was no difference in VO_2_max in women. Further statistical analysis attributed this sex difference to lower fitness levels and perhaps more importantly the lower muscle mass (~45 kg in women versus ~67 kg in men) in women participating in the study. The impact of fitness level on test duration suggests that the steeper ΔVO_2_/ΔWR slope in shorter protocols may be disadvantageous to individuals of lower fitness. This could be due to increased reliance on nonmitochondrial energy systems, thus causing premature fatigue, as well as eliciting central cardiovascular limitations.

## 5. VO_2_max Protocol Paradigm Shift

For the past 60 years the push to standardize GXT procedures has been essential to progress understanding of the complex and sensitive interaction between exercise and the integrated human physiological responses. In spite of the advancements that have given test administrators the ability to control fixed increments of intensities in an open-loop fashion (constant administrator testing variable manipulation without a fixed termination time), recent research has introduced an alternative approach to exercise protocols that allow the subject to self-pace the protocol in an incremental format [73–75]. In effect, this type of protocol would not negate past research that emphasized the role of the heart, lungs, circulatory, and other integrated systems in a limiting capacity but rather challenge the role of the brain as a potential simultaneous regulator. Although not entirely self-paced, studies by Pollock et al. [61, 76] used a model where speed was adjusted to accommodate individual movement efficiency and workload was increased by adding treadmill grade. In an effort to appreciate the evolution of self-paced protocols, the Pollock et al. [61, 76] investigations underscore the importance of identifying movement speeds that engage the greatest amount of muscle mass. Hagerman [77] was the first to report the ability of an individual to reach a greater VO_2_ by self-pacing during a simulated competitive time trial compared to laboratory-based testing methods. The finding suggested that an individual's ability to self-regulate muscular power output may serve as the ultimate variable in maximizing physiologic responses. Intrigued by this finding, Foster et al. [78] compared the physiological responses between a self-paced laboratory 5-km cycle time trial and a GXT using a cycle ergometer. They found that maximal VO_2_, HR, ventilation, and blood lactate (BLa^−^) levels were significantly greater in the 5-km time trial compared to the cycle ergometer GXT. These initial findings raised an important fundamental question regarding exercise testing protocols: if an individual possesses the ability to achieve a greater physiological ceiling when self-paced, does the search for achieving standardized protocol procedures serve a pragmatic purpose?

Eston and Thompson [73] used a closed-loop (no constant administrator manipulation and a fixed termination time) perceptually-regulated protocol guided by the 6–20 Rating of Perceived Exertion Scale (RPE) [79] to estimate maximal work rate in patients receiving *β*-blocker treatment. Using 4 × 3 min stages at an RPE of 9 (“very light”), 13 (“somewhat hard”), 15 (“hard”), and 17 (“very hard”), they reported that RPE could be used to predict maximal functional capacity. Similar RPE protocols using various stage lengths (2, 3, and 4 min) have been validated to accurately predict VO_2_max [74, 80–83]. Using a similar perceptually regulated paradigm, Mauger and Sculthorpe [75] investigated a self-paced cycle exercise protocol in 16 untrained university students. The test design consisted of 5 × 2-min stages, totaling 10 min, at incremental intensities utilizing Borg's Rating of Perceived Exertion (RPE_6–20_) Scale [79]. The protocol was designed as follows: stage one was clamped at an RPE of 11 (“fairly light”), stage two clamped at an RPE of 13 (“somewhat hard”), stage three clamped at an RPE of 15 (“hard”), stage four clamped at an RPE of 17 (“very hard”), and the final stage clamped at an RPE of 20 (“maximal exertion”). The unique aspect of the protocol was the additional stage of “maximal exertion” (RPE 20) to the established series of 2 min RPE-clamped stages as applied previously [80] in order to directly measure VO_2_max. The participants achieved a significantly greater VO_2_max (40 ± 10 versus 37 ± 8 mL·kg^−1^·min^−1^) and peak power output (273 ± 58 versus 238 ± 55 Watts) in the self-paced protocol compared to a traditional GXT despite the absence of significant differences in HRmax, RERmax, VEmax, and mean power output. It is important to note that the results of Mauger and Sculthorpe [75] have received considerable criticism over methodology. Their results have been attributed to discrepancies in test duration between self-paced (10 ± 0 min) and traditional (13 ± 3 min) protocols and that direct GXT protocol comparison must require a match in total test duration [84, 85]. Interestingly, the closed-loop nature of the test elicited a motivation or “final push” during the final stage of the test similar to that expected toward the end of an athletic competition. Moreover, the results support the role of the brain during a closed-loop setting when the individual is able to vary work rate constantly, balancing discomfort with a maintainable power output and willingness to complete the test. The simplicity of the protocol design has produced many speculative explanations for the results. Mauger et al. [86] showed that a speed-based self-paced treadmill test elicited significantly greater VO_2_max and HRmax values compared to those relative to a traditional test. This study also received criticisms over flawed methods and lack of control, attributing findings to using different modes (motorized versus nonmotorized treadmill) and neglect of measurement error to test their hypothesis [83, 87–89]. Follow-up studies have shown a higher VO_2_max attainment during self-pacing using a cycle ramp protocol [90], similar VO_2_max attainment using motorized treadmill [83, 91–93] and cycle protocols [84, 94], and a lower VO_2_max attainment using an automated treadmill [89]. It is important to note that findings showing no difference between self-paced and traditional protocols demonstrate the potential utility for self-paced GXT protocols, particularly when considering protocol duration. While test duration is tightly controlled during self-paced testing and the incremental steps in oxygen cost between stages have been shown to fall within recommended guidelines (1-2 METS) [95], physiological measurements that may distinguish self-paced from traditional protocols have yet to be adequately examined. It is purported that underlying variables that comprise the Fick equation, namely, *Q* and a-vO_2diff_, and the role of blood flow redistribution may underpin differences between self-paced and traditional protocols [92, 93]. More recently, Astorino et al. [90] showed that a self-paced cycle protocol elicited higher VO_2_ compared to a ramp protocol (50.2 ± 9.6 versus 47.2 ± 10.2 mL·kg^−1^·min^−1^). Additionally, they were the first to compare central cardiovascular responses between protocols and showed a higher *Q*_max_ during self-paced compared to ramp (21.9 ± 3.7 versus 20.7 ± 3.4 L·min^−1^). It should be noted that their average test duration was not tightly controlled to 10 min (9.6 ± 0.8 min); therefore pacing was not restricted throughout the final 2-minute stage. Although initial results are intriguing, the investigation into the efficacy and suitability of self-paced protocols is in its infancy. Therefore, future researchers could choose to design studies to expound on the intertrial reliability using self-pacing protocols. Additionally, studies should examine the interaction between central, peripheral, and central regulating responses during self-paced exercise.

## 6. VO_2_max Attainment Criteria

In order to increase the reliability and validity of a test, an undefined combination of standardized criteria must be met during the GXT including the following: VO_2_ plateau, estimated HRmax, RER, BLa^−^, and RPE. This widely accepted set of characteristics, or VO_2_max criteria, has become a controversial topic of debate in recent years due to the high intersubject variability in attaining the criteria [96–99]. Furthermore, the number and type of criteria used to determine VO_2_max are often contingent on the preference of the researcher or clinician administering the test [96]. Along with protocol design, other factors such as metabolic data processing methods and participant effort make comparing the results for clinical or research purposes difficult [53, 99, 100].

## 7. Detection of a VO_2_ Plateau

As with many of the principles used today in exercise physiology, the original reports of a slowing or “plateau” of oxygen consumption despite increasing muscular work can be attributed to Bassett Jr. and Howley [101]. Taylor et al. [53] later confirmed the existence of a VO_2_ plateau in 9 of 13 men during a treadmill test of incremental speed (increasing 1 mph) and 108 of 115 (94%) men during treadmill tests of incremental grade (2.5%). Studies have since demonstrated that a VO_2_ plateau can be detected in 17% to 100% of subjects tested, suggesting that its existence represents an inconsistent “phenomenon” [100]. Taylor et al. [53] were the first to apply the VO_2_ plateau criterion of ≤150 mL·min^−1^, defined by a change in VO_2_ ≤150 mL·min^−1^ despite a continuous increase in workload. This value, alongside the wide range of other values used as plateau criteria (≤50 mL·min^−1^, ≤100 mL·min^−1^, ≤200 mL·min^−1^, and ≤280 mL·min^−1^), should not be applied universally since the criteria must reflect the expected rate of VO_2_ increase per unit time relative to the protocol design [102]. Among the most important factors impacting the incidence of a VO_2_ plateau are age [103], testing modality [104], and data analyses methodology [99, 105]. Astorino et al. [103] showed that the strongest predictor of VO_2_ plateau among 30 men and women consisting of groups of endurance-trained (*n* = 9), recreationally active (*n* = 11), and strength trained (*n* = 10) individuals was age, not training status, body composition, or training history. Gordon et al. [104] demonstrated that treadmill testing (58%) was superior to cycle ergometer testing (8%) at eliciting a plateau. The diminished plateau in cycling was attributed to the increased metabolic cost of the eccentric skeletal muscle activity in treadmill running compared to the concentrically dominant cycle exercise. Furthermore, Astorino [105] confirmed that gas sampling rate impacted the incidence of a plateau. In this study, 13 sedentary, 48 recreationally active, and 47 competitive athletes completed a GXT using treadmill and cycle protocols. The group found that the incidence of plateau was greater using breath-by-breath (81%), 15-sec (91%), and 30-sec (89%) averaging compared to a longer sampling rate of 60-sec (59%). Collectively, these findings suggest that sampling rate in conjunction with the plateau threshold criteria may explain much of the discrepancy in incidence of VO_2_ plateau across studies.

### 7.1. Heart Rate Response to the VO_2_max Protocol

Due to its noninvasive nature, simplicity, and fairly predictable response to incremental to maximal exercise, HR is often used as a secondary criterion to VO_2_ plateau. Much like plateau and other secondary criteria, the criteria for HR are highly variable. Typically, the threshold is established at a specific percentage using an age-predicted HRmax equation. Surprisingly, Fox et al. [106] created the 220-age equation by drawing an arbitrary best fit line from the observation of 10 studies [106]. Robergs and Landwehr [107] evaluated the Fox equation [106] and highlighted the fact that there were no statistical methods used to establish the regression equation from their data set. Instead, Fox and colleagues [106] summarize their methods by stating that “no single line will adequately represent the data on the apparent decline of HRmax with age. The formula, 220-age, defines a line not far from many data points.” Curious to investigate the mystery of 220-age, Robergs and Landwehr [107] replicated the data set presented by Fox et al. [106], applied linear regression analysis, and found the equation to be 215.4–0.9147 (age) with a ±21 bpm error. Tanaka et al. [108] cross-validated an age-predicted HRmax equation by combining 351 studies (18,712 subjects) and a laboratory investigation using 514 subjects (18–81 years old). Their regression analysis established a new equation (208 − 0.7 × age) with a less, yet still substantial (±7–11 bpm), error range. It was concluded that the 220-age equation underestimates age-predicted HRmax in individuals over the age of 40 years. For these reasons, equations with less inherent error should be applied when using “±10 bpm of age-predicted HRmax” as a secondary VO_2_max criterion.

### 7.2. RER Implication of the VO_2_max Protocol

The RER is another secondary VO_2_max criterion that is used to reflect the balance between bicarbonate buffering and hydrogen ion accumulation in the face of incremental exercise. Upon increasing metabolic acidosis, bicarbonate buffering leads to an increase in CO_2_ production, increased ventilation, and a subsequent increase in RER. A series of studies by Issekutz et al. [109, 110] was the first to examine the relationship between RER and incremental exercise, VO_2_max, and the metabolic state of the exercising human. Using a series of 4-5 min intermittent maximal exercise tests, they calculated the difference between CO_2_ and the product of a metabolic respiratory quotient constant and VO_2_ (CO_2_  − VO_2_  × 0.75). This value was termed “excess CO_2_” and used to reflect the change in substrate utilization and exercise intensity with the increase in VO_2_ [109, 110]. Ultimately, they established the most widely used threshold for RER criteria (≥1.15) today. Much like HR and VO_2_plateau criteria, a standard RER threshold value is not consistently applied, using 1.0, 1.05, 1.08, 1.10, 1.12, 1.13, or 1.20 to verify VO_2_max attainment [96, 97]. The high intersubject variability in RER responses due to inconsistent effort levels and training status makes higher RER values difficult to achieve for many individuals [97].

### 7.3. VO_2_max and Blood Lactate Accumulation

Analysis of postexercise BLa^−^compared to preexercise BLa^−^ has been used as a reliable marker to quantify exercise intensity. Hill et al. [4–6] were the first to establish a relationship between BLa^−^ and exercise intensity during vigorous to maximal exercise. Regardless of the debate for practical application or metabolic circumstances, it is agreed that BLa^−^ accumulation is related to an individual's ability to tolerate and sustain exercise; therefore, it is commonly used as a surrogate measure of the metabolic perturbations during maximal exercise and a secondary VO_2_max criterion [111, 112]. The origins of the criterion date back to an investigation by Astrand [113] who used postexercise BLa^−^ concentrations to verify VO_2_max in young (14–18 years) boys and girls. Despite the fact that only half of the subjects demonstrated a VO_2_ plateau, it was noted that the individuals who exhibited a plateau had postexercise BLa^−^ concentrations between 7.9 and 8.4 mM (average ≥ 8 mM). It has since been determined that factors such as age, training status, sex, and overall effort may impact the level of BLa^−^. This has led to the use of a ≥10 mM threshold or even a complete disregard for maximal BLa^−^ concentration due to the high intersubject variability (anywhere from 1.2 to 18 mM) in postexercise lactate [96, 98, 114].

### 7.4. RPE Assessment in a VO_2_max Protocol

The simplest and most controversial measurement traditionally used as a VO_2_max criterion was developed by Borg [79], known as Borg's Rating of Perceived Exertion (RPE) Scale_6–20_. While RPE is not a direct measurement of physiological responses, the behavioral, motivational, and physical factors that an individual perceives during GXT contribute greatly to the overall validity of the test. Many studies have shown a strong relationship between RPE, HR, and VO_2_ [82, 115–117]; however, others have demonstrated RPE to be less related to these variables in less active or sedentary individuals [118, 119]. Interestingly, Noakes [120] suggested that thresholds may not be as simple as limitations in central and peripheral components but rather controlled by a “central governor” that regulates self-pacing and overall effort throughout various points of a maximal exercise bout. Despite the relationship among RPE, HR, and BLa^−^, inconsistencies in recent studies call to question the validity of RPE. Edvardsen et al. [114] examined the attainment of the commonly used RPE criterion of ≥17 in 840 individuals (20–85 years) and found that 84% of the subjects were able to achieve the criterion. Due to the variability and subjective nature of the criterion, Magnan et al. [121] examined the attainment of an RPE ≥18 in 240 inactive individuals (18–45 years) and found that 93.7% of the individuals reached the desired RPE threshold despite the fact that only 59% were demonstrating a VO_2_ plateau. Overall, the assumptions in employing an RPE criterion depend on the subject's understanding of the scale and associated verbal descriptors, ability to differentiate between discomfort and physiological fatigue, and motivation.

## 8. Verification Protocols of VO_2_max

Many of the current criteria used to determine VO_2_max were established with technology that is no longer used today. Douglas bags and Tissot gasometers have been replaced by sophisticated metabolic analyzing systems and pneumotach and turbine flow measurement devices. Furthermore, the VO_2_max criteria were developed using certain modalities (treadmill versus cycle) on relatively small samples of homogenous populations. These reasons and the overall variability of the criteria have resulted in some researchers rejecting secondary criteria altogether. Recent attempts have been made to establish new VO_2_max attainment criteria through the introduction of a verification protocol [97, 122–126]. The origin of the verification protocol is believed to exist in a text by Thoden et al. [127], in which the first recommendation was made to include a bout of supramaximal exhaustive exercise (higher workload than achieved during GXT) following the completion of GXT. Since the inception of the verification protocol, there has been much effort to establish verification protocol intensity, duration, rest period after completing initial GXT, and the criteria used to verify VO_2_max attainment. In follow-up guidelines, Thoden [128] recommended that the recovery phase between GXT and verification protocol should be between 5 and 15 minutes and a workload of one stage higher than the final completed stage during GXT should be used. Unfortunately, the recommendations by Thoden et al. [127, 128] were theoretical rather than research-based guidelines. The first study to directly examine the efficacy of a verification protocol was done by NiemelÄ et al. [129], who performed a verification protocol at a workload equal to the greatest workload achieved during initial GXT within a week of completing initial GXT. Using a ±5% repeatability range for VO_2_max [130], they were able to confirm attainment in 8 of the 16 subjects. Day et al. [8] sought to observe the differences between cycle GXT and a subsequent verification test in 38 healthy individuals (19–61 years) and reported no difference in VO_2_max using a workload of ~90%  *W*_peak_. Follow-up studies commonly used verification workloads between 95 and 130% of peak workload achieved during initial cycle GXT or 0.5–1.6 km·hr^−1^ higher than the peak velocity achieved during initial treadmill GXT. The recovery time between tests ranged from the same day [122, 126, 131–134], 1–10 min active rest [122, 132, 133, 135], and 5–60 min passive rest [125, 135], to separate day testing [8, 123, 126]. Furthermore, tests were done on endurance-trained runners [122, 123], male athletes [135], recreationally active men and women [122, 125, 126], sedentary men and women [105], and middle-aged men and women [131]. Studies commonly revealed a nonsignificant mean difference in VO_2_max between GXT and verification protocols when analyzed on an individual, rather than group mean, basis within the ±5% measurement error (accuracy of metabolic system when properly calibrated according to manufacturer) associated with VO_2_ measurement [136–138]. Most recently, a study by Nolan et al. [125] investigated the impact of two verification intensities and rest periods. After an initial treadmill test, 12 active males and females completed each of the following four verification conditions: 105% maximal GXT workload, 20 min rest; 105% maximal GXT workload, 60 min rest; 115% maximal GXT workload, 20 min rest; and 115% maximal GXT workload, 60 min rest. Their results demonstrated a 100% success rate in verifying attainment of VO_2_max when using 105% maximal GXT workload, regardless of the rest period between tests, and highlighted the current recommendation for intensity and rest period optimization during verification tests [125].

## 9. Error in VO_2_max Measurement

The sophisticated metabolic systems used to collect and analyze data during exercise testing represent the most sensitive and reliable means for laboratory-based research. In order to compare results between studies, the data must be validated. Even if metabolic systems are appropriately maintained and calibrated, measurement error of ±5% is commonly accepted [136–138]. A study by Yule et al. [139] reported a 15% difference in VO_2_max between three identical systems in the same laboratory. Furthermore, differences attributed to measurement error between 10 and 22% have been reported when comparing identical testing protocols using different metabolic systems [137]. A meta-analysis by Vickers [140] examined the test-retest reliability in maximal exercise testing and found that the average standard measurement error was 2.58 mL·kg^−1^·min^−1^. To account for total error, however, a source of biovariation reflecting the inherent biological fluctuations within an individual must also be considered [141]. Knowing that the underlying assumption for comparing VO_2_max between and within studies is that biological variability must account for a portion of total error, Katch et al. [141] designed a study in which five participants completed an average of 16 maximal exercise tests over the course of a 2–4-week period. They found that, within the total error of ±5.6%, biological variability accounted for ~93% of the error while measurement error accounted for only ~7%. Meanwhile, recommendations by Balady et al. [138] report that the biological component of variability intrinsic to GXT is commonly accepted within 3-4%. Due to the numerous factors that contribute to biological variability and subsequent total error, an argument can be made against studies that consider only manufacturers' guidelines of measurement error when comparing tests [142]. This can be represented by a scenario comparing VO_2_ responses to various protocol designs, whereas an individual who falls within the accepted measurement error range between tests may have only done so based on the small contribution in variance from the system rather than the large contribution from biological variability due to heredity, homeostatic stress, training status, psychological stress, sleep, or nutrition [143]. For this reason, comparing attainment of VO_2_max between protocols may be more reliable when considering total error (biological variability + measurement) rather than measurement error alone.

## 10. Conclusion

Due to the valuable information gathered and the wide spectrum of applications for the use of GXT, it has become an increasingly important objective to derive an optimal set of standardized procedures for the determination of VO_2_max. Many years of observations examining the potential sources for individual variability in GXT responses are cited in the literature. Despite the pitfalls in physiological variability, standardized tests using traditional methods for VO_2_max verification remain the most commonly employed. Furthermore, the methods in which these criteria have been previously established, as well as the comparison between studies evaluating the appropriateness of universal protocols, do not consider combined sources of inherent measurement and biological error. These reasons underpin the current suitability of more standardized GXT guidelines and subsequent methods for determining test validity. More recent approaches have highlighted alternative methods for measuring exercise capacity using a closed-loop, self-paced testing model. Future research directions should seek investigating perceptually regulated (RPE-clamped) protocols with verification protocols for the overall suitability and individualization of GXT.
